# A Rare Case of Combined Langerhans Cell Histiocytosis and Adult-Onset Xanthogranuloma: A Case Report

**DOI:** 10.7759/cureus.28640

**Published:** 2022-08-31

**Authors:** Yash D Bhavsar, Abhishek V Dudhatra, Himali S Vyas, Rushikesh B Pandya, Arpit P Joshi

**Affiliations:** 1 Internal Medicine, Smt. Nathiba Hargovandas Lakhmichand (NHL) Municipal Medical College, Ahmedabad, IND; 2 Oncology, Singapore General Hospital, Singapore, SGP; 3 Hematology and Oncology, Healthcare Global Enterprises (HCG) Cancer Centre, Ahmedabad, IND; 4 Internal Medicine, Byramjee Jeejeebhoy (BJ) Medical College, Ahmedabad, IND

**Keywords:** xanthogranuloma, langerhans cell histiocytosis (lch), langerhans cell histiocytosis, non-langerhans histiocytosis, adult xanthogranuloma, juvenile xanthogranuloma, oral cavity squamous cell carcinoma

## Abstract

Langerhans cell histiocytosis (LCH) and adult-onset xanthogranuloma (AXG) are rare disorders characterized by the accumulation of macrophage, dendritic cells, or monocyte-derived cells in various tissues of the body. Many researchers now consider LCH a form of malignancy, but this classification remains controversial. As per our knowledge, there are only 36 cases of AXG reported so far in the English literature. Here, we report a case of AXG and single-system LCH found in the oral cavity and cervical lymph nodes, respectively. In this article, we intend to define a clear understanding of some classic clinical, radiological, and histopathological findings of LCH and AXG, to differentiate them from oral malignancies. The primary goal of this article is to increase awareness regarding conditions that closely resemble malignancies and to save patients from the burden of extensive treatment under the presumption of malignant disorders. In the medical field, reporting of rare cases is highly encouraged; however, proper treatment for the patient depends on the accurate diagnosis that, in this case, was made postoperatively, which only added more physical and mental distress for the patient and their family.

## Introduction

Langerhans cell histiocytosis (LCH) and adult-onset xanthogranuloma (AXG) are two separate forms of histiocytic disorders characterized by abnormal proliferation of dendritic cells, lymphocytes, plasma cells, macrophages, and neutrophils. Oral squamous cell carcinoma (OSCC) is a malignant condition that shows a relatively poor prognosis when compared to AXG and LCH [1]. Histiocytic disorders are classified into five groups of diseases: (i) Langerhans-related, (ii) cutaneous and mucocutaneous, (iii) malignant histiocytosis, (iv) Rosai-Dorfman disease, and (v) hemophagocytic lymphohistiocytosis and macrophage activation syndrome. The incidence of LCH has been reported to be 0.5-5.4 cases per million persons per year, according to prospective registry-based reports [2]. LCH is characterized by the presence of CD1a(+) and S100(+) markers, while AXG is characterized by the presence of CD68(+) cells [3]. Juvenile xanthogranuloma (JXG) is the most common form of non-Langerhans cell histiocytic disorder in childhood and rarely is found in adults as AXG, with a peak incidence in the late 20s to 30s [4]. The majority of adult patients have solitary lesions [5]. The true incidence, however, may be underestimated, as many lesions, especially those which are solitary and small (up to 90% of all patients), may go unrecognized. It can occur anywhere in the body, commonly including bone, spleen, liver, lymph nodes, and CNS, but oral lesions are comparatively rare that may ulcerate and bleed [6]. As per our knowledge, there are only 36 cases of AXG reported so far in the English literature, out of which only eight cases involved the oral cavity [7]. We report the first case of non-bone, buccal mucosal AXG in a 58-year-old man in India.

AXG of the oral cavity could be misdiagnosed with malignancies like OSCC due to the presence of typical ulcerative lesions. The presence of such ulcerative lesions in the oral cavity could potentially represent benign conditions like pseudoepitheliomatous hyperplasia (PEH), which is often confused for OSCC due to their histologic similarities. Incisional biopsy of the PEH lesion would show hyperplasia and acanthotic squamous cells, resembling a well-differentiated OSCC [8]. Some commonly known causes of PEH lesions include infection, chronic inflammation, and mechanical or chemical tissue injury [8]. Other than PEH, mucosal keratoacanthoma and inverted follicular keratosis are some other benign lesions that could mimic OSCC. Biopsy and histopathological examination are necessary for accurate diagnosis. However, physicians should exercise concern not to miss a malignant pathology that could lead to metastases and death.

In our case, after an initial diagnosis of OSCC, a postoperative immunohistochemical analysis of the excisional frozen specimen was performed and the diagnosis was changed to AXG of right buccal mucosa and LCH of right-sided level 1a, 1b, and level 2 cervical lymph nodes. This article intends to enable oncologists, surgeons, hematologists, and pathologists to collaborate and better differentiate the two conditions clinically.

## Case presentation

This observational case report presents a 58-year-old Indian male patient with a chief complaint of one month of painless right-sided cheek swelling of up to 3 cm, which remained stable in size since the patient first noticed it. He also had a single painless episode of oral mucosal bleeding provoked by brushing about three weeks earlier. The patient has had a history of hypertension for six years, controlled by dual antihypertensive agents. The patient smoked half-pack a day for 20 years and documented no history of alcohol consumption. The patient was retired but had an active lifestyle and played medium-intensity sports for two hours a day. He denied having fevers, chills, night sweats, and weight loss. Family history was positive for OSCC, diagnosed in an elder brother at the age of 57. After an initial examination by an internist, a consultation with an ENT surgeon was scheduled. Upon initial physical examination, two most important findings were documented: (i) right-sided cheek swelling, which was mobile, soft, and without adjacent palpable cervical or submandibular lymph nodes, and (ii) incidental finding of about 2 cm painless, irregular verrucous appearing ulceration in the right buccal mucosa above the second and third molars of the right lower jaw. Fine-needle aspiration cytology (FNAC) of the right cheek swelling and an incisional biopsy of the right buccal mucosal lesion were performed. A contrast-induced MRI of the head and neck was also ordered, which showed a well-defined lesion (measuring 2.2 (w) x 1.7 (anteroposterior) x 1.6 (craniocaudal) cm) in the right buccal mucosa at the level of the occlusal plane. It showed central necrosis and peripheral enhancement along with mild adjacent edema representing a possible infective process or malignant neoplastic node involvement simultaneously. Mildly enlarged non-necrotic nodes at “right level Ib” and two other non-necrotic nodes at right “level Ia” were reported as suspicious for malignancy by the radiologist.

An incisional biopsy of the ulcerative lesion at the right buccal mucosa was collected and sent for histopathology, which returned positive for OSCC. The FNAC cytology report showed a “large number of neutrophils and few lymphocytes and foamy macrophages in a necrotic background.” A combined diagnosis of well-differentiated squamous cell carcinoma (OSCC) of the right buccal mucosa with localized right-sided cheek abscess was documented. The patient was further referred to a surgical oncologist, and after the initial examination and biopsy report analysis, a provisional diagnosis of OSCC was decided and explained to the patient. He was advised to undergo a whole-body positron emission tomography-computed tomography (PET-CT) scan for definitive staging and initial screening for any metastasis.

**Figure 1:**
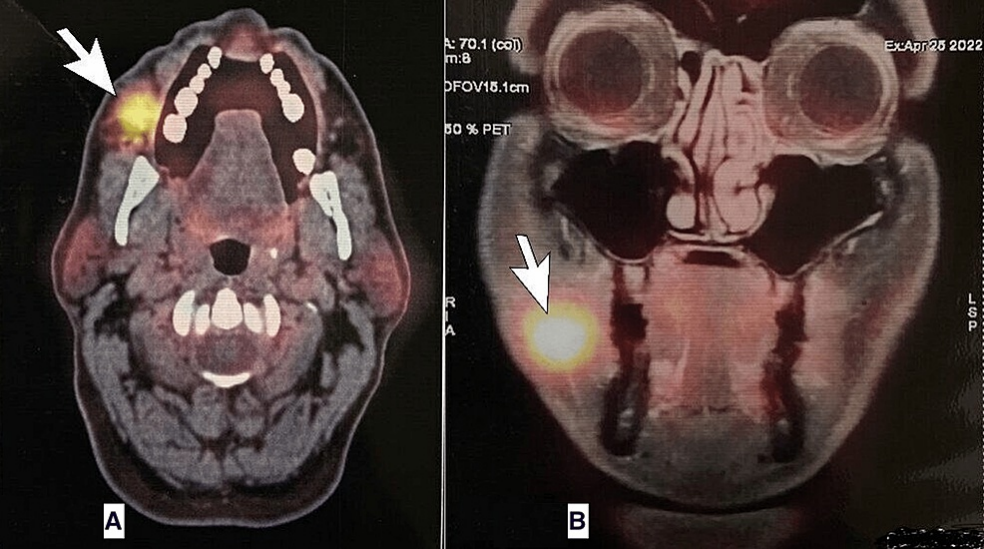
Positron emission tomography-CT with fusion MRI scan of the head showing fluorodeoxyglucose-avid lesions in right buccal mucosa marked by white arrows

PET-CT scan with MRI fusion study was performed, which showed metabolically active heterogeneously enhancing lesion involving the right buccal mucosa and lower gingivobuccal sulcus (Figure 1). Non to low-grade metabolically active right-level IA, right-level IB nodes, and a few small nodes along right facial vessels were noted suspicious of metastasis. There was no evidence of distant body metastasis. Further, the oncologist discussed a surgical plan for radical dissection of the right cheek along with right level 1 and 2 cervical nodes with the patient, and after appropriate consent, the procedure was performed.

**Figure 2:**
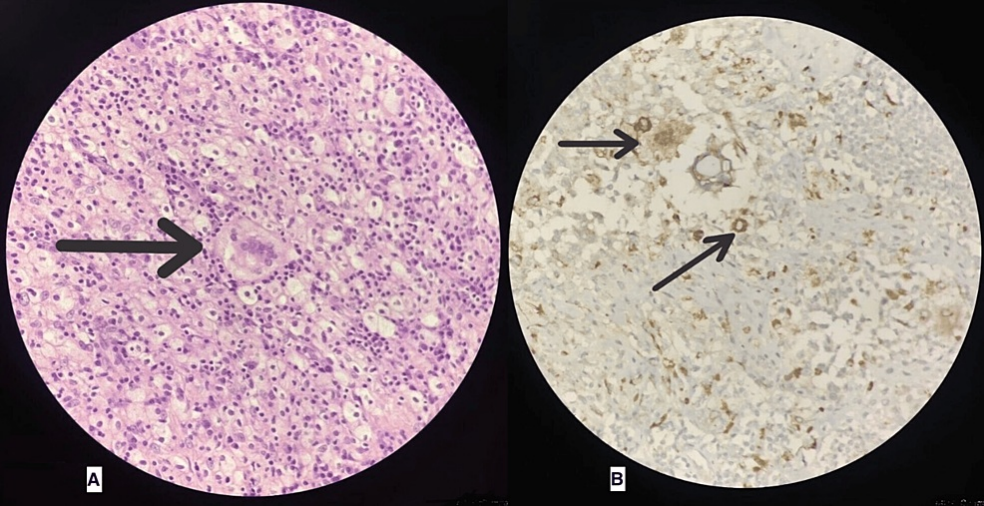
Immunohistochemistry of buccal mucosa specimen showing "Touton giant cells" (A) and "CD68 cells" (B) consistent with adult-onset xanthogranuloma

**Figure 3:**
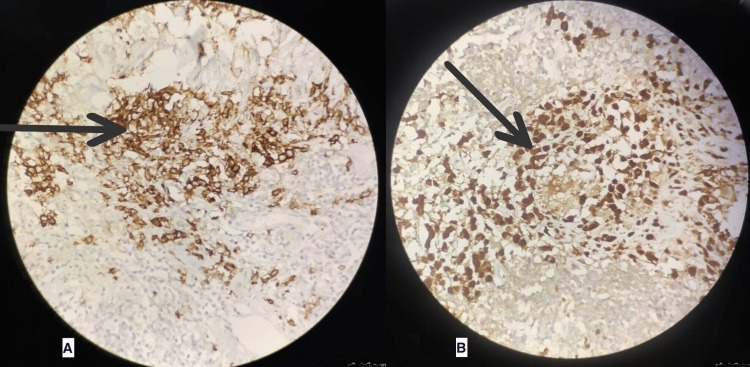
Immunohistochemistry of cervical lymph node specimen showing "CD1A" cells (A) and "S-100" cells (B) consistent with Langerhans cell histiocytosis

The postoperative course was uneventful, with expectant surgical wound healing without any other major or minor complications. Four weeks after surgery, the excisional biopsy and immunohistochemical results of the excisional specimens came back positive for a diagnosis of AXG in the buccal mucosa (Figure 2) and LCH in level 1a and 1b cervical nodes (Figure 3). The patient was explained about the rare occurrence of this disorder and the presence of a poorly understood management plan, especially in adults. The patient was referred to a hemato-oncologist, who described no need for further treatment as surgical removal is curative and had already been performed in this case. The patient was scheduled for a follow-up after four months.

## Discussion

LCH in adults is infrequently reported and is known to predominantly affect the pediatric population. Several reports approximate that 90% of patients who present with localized skeletal LCH are between five and 15 years of age, with an average age of onset of 10-14 years [9,10]. LCH and AXG are rare forms of histiocytic disorders that present with a broad range of clinical presentations and are classified into five main groups as described above in this article [11]. Our case was identified as having combined “Type C” and “Type L” by the hemato-oncologist. The Histiocyte Society has published a revised classification in which LCH is sub-classified according to the site of manifestation and organ involvement: single system LCH, lung LCH, and multi-system LCH [12]. Single-system LCH (SS-LCH) most commonly affects the skin and bone, while other rare sites of involvement include the lungs, lymph nodes, and the central nervous system. The diagnosis is usually established by biopsy and immunohistochemistry of the lesion, which shows abundant eosinophils, foamy macrophages, and a characteristic presence of Touton giant cells that have a wreath-like arrangement of nuclei [13]. Detection of Langerhans cell markers (CD1a and S-100) is mandatory to confirm the diagnosis of LCH [14].

AXG is a rare, inconsistently defined non-Langerhans cell (NLC) disorder occurring in adults. Although the pathogenesis of AXG has not been established, it is believed that reactive processes in response to the initiating stimuli, possibly infectious or physical factors, may play a role in the development of AXG in adults [15]. In our case, several clinical and radiological findings, such as (i) history of tobacco use, (ii) typical buccal oral ulceration, (iii) mass in the right cheek, (iv) positive PET-CT of the lesion, and (v) family history of OSCC, pointed toward the diagnosis of OSCC, which was later confirmed by the histopathology report of the biopsy sample. However, minimal attention was given to the FNAC performed on the right cheek mass, which showed the presence of abundant neutrophils and foamy macrophages, suggesting a diagnosis of inflammatory abscess in the right cheek [13]. The presence of an inflammatory abscess close to OSCC lesions is unlikely and should provoke the physician to have a broader differential diagnosis before considering malignancy [16].

Furthermore, a positive fluorodeoxyglucose uptake on a PET-CT scan does not always reliably indicate malignancy in every case but can also be associated with other metabolically active conditions like a focus of chronic inflammation and infection in the body that consists of activated macrophages and neutrophils [17]. The underlying clinical, pathological, and radiological similarities between OSCC and LCH/AXG confounded the diagnosis of combined histiocytosis, which led to extensive radical surgery under the hood of presumed oral malignancy like OSCC. It is not uncommon for histiocytosis to be misdiagnosed as malignancy [18]. Therefore, a structural and sensitive approach to exclude other benign conditions (like LCH and AXG) is essential before suggesting a diagnosis of malignancy.

## Conclusions

Rare entities like LCH or AXG are challenging to diagnose and differentiate from malignancies like OSCC, especially when it presents in an adult age group along with a positive history of confounding factors like increased age (>50 years old), tobacco use, typical appearance and location of the ulcer, and positive family history. Appropriate immunohistochemical analysis of the biopsy sample taken for suspected malignancy holds colossal value in defining and differentiating rare disorders like LCH and AXG. Under false pretenses of malignancies, radical surgeries are often performed, which gravely affect the patient's quality of life. As described in this article, proper clinical correlation of history and other presenting signs could allow physicians to have a relatively higher threshold for diagnosing malignancies. OSCC is the most common tumor type diagnosed in patients with oral cavity lesions, while LCH and AXG are rare. Hence, it is imperative to diagnose and treat life-threatening malignancies appropriately while staying alert for rare diseases as part of differential diagnoses.

## References

[1] Markopoulos AK (2012). Current aspects on oral squamous cell carcinoma. Open Dent J.

[2] Kim H, Kim YJ, Shin J (2019). Analysis of the incidence and diagnostic pattern of Langerhans cell histiocytosis from the population-wide healthcare database in Korea. Blood.

[3] Ishikawa M, Endo Y, Uehara A, Suto M, Yasuda M, Motegi SI, Ishikawa O (2019). Cutaneous adult xanthogranuloma with a small portion of BRAFV600E mutated Langerhans cell histiocytosis populations: a case report and the review of published work. J Dermatol.

[4] Chen CY, Sung CL, Hsieh MY, Wang WC, Lin LM, Chen YK (2015). An adult juvenile xanthogranuloma in the buccal mucosa. J Dent Sci.

[5] Numbere N, Pukhalskaya T, Bowman B, Campbell K, Smoller B (2021). Progressive nodular histiocytosis: report of a case and review of the literature. Case Rep Pathol.

[6] Flaitz C, Allen C, Neville B, Hicks J (2002). Juvenile xanthogranuloma of the oral cavity in children: a clinicopathologic study. Oral Surg Oral Med Oral Pathol Oral Radiol Endod.

[7] Popa LG, Mihai MM, Orzan OA, Beiu C, Tebeica T, Giurcaneanu C (2019). Adult onset xanthogranuloma - case report and review of literature. Ro Med J.

[8] Sarangarajan R, Vedam VK, Sivadas G, Krishnaraj R, Sarangarajan A, Shanmugam KT (2015). Pseudoepitheliomatous hyperplasia: relevance in oral pathology. J Int Oral Health.

[9] Howarth DM, Gilchrist GS, Mullan BP, Wiseman GA, Edmonson JH, Schomberg PJ (1999). Langerhans cell histiocytosis: diagnosis, natural history, management, and outcome. Cancer.

[10] Stull MA, Kransdorf MJ, Devaney KO (1992). Langerhans cell histiocytosis of bone. Radiographics.

[11] Emile JF, Abla O, Fraitag S (2016). Revised classification of histiocytoses and neoplasms of the macrophage-dendritic cell lineages. Blood.

[12] Luz J, Zweifel D, Hüllner M, Bühler M, Rücker M, Stadlinger B (2018). Oral manifestation of Langerhans cell histiocytosis: a case report. BMC Oral Health.

[13] Kumar N, Sayed S, Vinayak S (2011). Diagnosis of Langerhans cell histiocytosis on fine needle aspiration cytology: a case report and review of the cytology literature. Patholog Res Int.

[14] Uppal P, Bothra M, Seth R, Iyer V, Kabra SK (2012). Clinical profile of Langerhans cell histiocytosis at a tertiary centre: a prospective study. Indian J Pediatr.

[15] Soilleux EJ (2010). Recent advances in mastocytosis and neoplasms of probable monocytic/dendritic cell lineage. Diagn Histopathol.

[16] Bolesina N, Femopase FL, de Blanc SAL, Morelatto RA, Olmos MA (2012). Oral squamous cell carcinoma clinical aspects. Oral Cancer.

[17] Tan GJ, Berlangieri SU, Lee ST, Scott AM (2014). FDG PET/CT in the liver: lesions mimicking malignancies. Abdom Imaging.

[18] Gonçalves CF, Morais MO, de Cássia Gonçalves Alencar R, Batista AC, Mendonça EF (2016). Solitary Langerhans cell histiocytosis in an adult: case report and literature review. BMC Res Notes.

